# Supplementary material for: Empirical methods for controlling false positives and estimating confidence in ChIP-Seq peaks
Source: BMC Bioinformatics. 2008 Dec 5;9:523. doi: 10.1186/1471-2105-9-523 (PMC2628906; doi:10.1186/1471-2105-9-523)
Supplement: Additional File 1 — A variety of html documents from the USeq web site detailing the available applications, their best usage, output file type descriptions, command line menus, etc. [file 1471-2105-9-523-S1.zip › USeqUserGuides/JavaDoc.zip!/JavaDoc/selex/SeqRead.html]

SeqRead


|  |  |  |
| --- | --- | --- |
| **Overview** \| **Package** \| **Class** \| **Use** \| **Tree** \| **Deprecated** \| **Index** \| **Help** | |  |
| **PREV CLASS**   **NEXT CLASS** | **FRAMES**    **NO FRAMES**     **All Classes** |  |
| SUMMARY: NESTED \| FIELD \| CONSTR \| METHOD | DETAIL: FIELD \| CONSTR \| METHOD |  |


---


## selex Class SeqRead

```
java.lang.Object
  selex.SeqRead
```

---

``` public class SeqRead extends java.lang.Object ```

Container for information related to a particular sequencing read.

---

**Constructor Summary**

`SeqRead(java.lang.String sequence,
java.lang.String seqComment,
java.lang.String quality,
java.lang.String qualComment,
SelexParams aSP,
SeqFiles aSF)`


| **Method Summary** | |
| --- | --- |
| `boolean` | `checkEstimateNumSubSeqs()` |
| `static boolean` | `checkSeqQuality(int[] qualityScores, int minNumHighQualBases, int minQualScore)`             Examines an int[] for a continuous stretch of scores |
| `int[][]` | `extractSegs(java.util.ArrayList ends)` |
| `java.util.ArrayList` | `findEnds()` |
| `static int` | `getNumStretchesGoodQuality(int[] qualityScores, int qualCutOff, int seqLength)`             Counts the number of stretches of scores above a cut off in an int[]. |
| `boolean` | `makeSubSeqs()` |
| `void` | `printSeqRead()` |
| `java.util.ArrayList` | `removeLowQualitySegs(int[][] segs)` |
| `java.util.ArrayList` | `removeOddSizedSegs(java.util.ArrayList segs)` |

**Methods inherited from class java.lang.Object**

`equals, getClass, hashCode, notify, notifyAll, toString, wait, wait, wait`

**Constructor Detail**

### SeqRead

```
public SeqRead(java.lang.String sequence,
               java.lang.String seqComment,
               java.lang.String quality,
               java.lang.String qualComment,
               SelexParams aSP,
               SeqFiles aSF)
```


**Method Detail**

### checkEstimateNumSubSeqs

```
public boolean checkEstimateNumSubSeqs()
```

---


### printSeqRead

```
public void printSeqRead()
```

---


### makeSubSeqs

```
public boolean makeSubSeqs()
```

---


### findEnds

```
public java.util.ArrayList findEnds()
```

---


### extractSegs

```
public int[][] extractSegs(java.util.ArrayList ends)
```

---


### removeLowQualitySegs

```
public java.util.ArrayList removeLowQualitySegs(int[][] segs)
```

---


### removeOddSizedSegs

```
public java.util.ArrayList removeOddSizedSegs(java.util.ArrayList segs)
```

---


### checkSeqQuality

```
public static boolean checkSeqQuality(int[] qualityScores,
                                      int minNumHighQualBases,
                                      int minQualScore)
```

:   Examines an int[] for a continuous stretch of scores

---


### getNumStretchesGoodQuality

```
public static int getNumStretchesGoodQuality(int[] qualityScores,
                                             int qualCutOff,
                                             int seqLength)
```

:   Counts the number of stretches of scores above a cut off in an int[]. Useful for estimating the
    number of concatinated selex oligos that should be found in a sequence read


---


|  |  |  |
| --- | --- | --- |
| **Overview** \| **Package** \| **Class** \| **Use** \| **Tree** \| **Deprecated** \| **Index** \| **Help** | |  |
| **PREV CLASS**   **NEXT CLASS** | **FRAMES**    **NO FRAMES**     **All Classes** |  |
| SUMMARY: NESTED \| FIELD \| CONSTR \| METHOD | DETAIL: FIELD \| CONSTR \| METHOD |  |


---
